# The Gardener's Dictionary

**Published:** 1834-01-01

**Authors:** 


*The Gardener's Dictionary.* By PHILIP MILLER, F.R.S.—London, 1833. Part I. 8vo. pp. 48; and 2 plates.

THIS is a still minuter fraction of a book, being only $\frac{1}{50}$. The fifty numbers are to be published within a twelvemonth; so that a stiff arithmetician would be ready to suppose that a number must come out every week—no such thing—the prospectus has the most profound contempt for such old-fashioned calculations; the numbers "will appear uninterruptedly on the 1st of every month, or oftener."

A little half-crown manual, giving a list of books in numbers which have never been completed at all, or not within the proposed time, or not for the proposed price, would be a very useful thing. We recommend some one of our youngest readers who is not yet "occupied with a multiplicity of engagements" (to use the medical phrase,) to undertake the compilation of this literary mentor; and we will pledge ourselves to buy a copy, *provided it does not come out in parts.*

